# Improvement of Cystic Fibrosis-Associated Liver Disease in Adults on Long-Term Cystic Fibrosis Transmembrane Conductance Regulator (CFTR) Modulators

**DOI:** 10.3390/life15121794

**Published:** 2025-11-24

**Authors:** Sofia Manioudaki, Larisa Vasilieva, Eleni Geladari, Iliana Mani, Zoe Athanassa, Ioannis Elefsiniotis, Emilia Hadziyannis, Vasilios Sevastianos, Aikaterini Oikonomou, Andreas Theophilou, Filia Diamantea, Alexandra Alexopoulou

**Affiliations:** 1 Intensive Care Unit, Sismanogleio General Hospital, 15126 Athens, Greece; manioudaki.sofia@gmail.com (S.M.); zathanassa@yahoo.gr (Z.A.); 2 Department of Gastroenterology, Alexandra General Hospital, 11528 Athens, Greece; larisatheo@yahoo.gr; 3 3rd Department of Internal Medicine and Liver Outpatient Clinic, Evangelismos General Hospital, 10676 Athens, Greece; elgeladari@gmail.com (E.G.); vsevastianos@gmail.com (V.S.); 4 2nd Department of Internal Medicine and Research Laboratory, Medical School, National and Kapodistrian University of Athens, Hippokration Hospital, 11527 Athens, Greece; ilianamani@windowslive.com (I.M.); emhadzi@med.uoa.gr (E.H.); 5 Hepatogastroenterology Unit, Academic Department of Internal Medicine, “Agioi Anargyroi” General and Oncology Hospital of Kifisia, National and Kapodistrian University of Athens, 14564 Athens, Greece; ielefs@nurs.uoa.gr; 6 3rd Department of Internal Medicine, Sotiria Thoracic Diseases Hospital, 11527 Athens, Greece; katherina.economou@gmail.com; 7 Medical School, National and Kapodistrian University of Athens, 11527 Athens, Greece; e1t32676trgg@gmail.com; 8 Adult Cystic Fibrosis Unit, Sismanoglio Hospital, 15126 Athens, Greece

**Keywords:** cystic fibrosis, cystic fibrosis-related liver disease, liver stiffness, CFTR modulators, lumacaftor/ivacaftor, elexacaftor/tezacaftor/ivacaftor, adults

## Abstract

Cystic fibrosis (CF) transmembrane conductance regulator (CFTR) modulators have been reported to improve lung function and reduce CF exacerbations. We aimed to investigate the efficacy of CFTR-modulators in CF-associated liver disease (CFLD) during long-term treatment. Longitudinal data were collected from genetically confirmed adult CF patients receiving CFTR-modulators. CFLD was diagnosed using conventional criteria combined with liver stiffness measurement (LSM). A total of 57 patients [56.1% male; median age at baseline (T0), 26 years (interquartile range [IQR], 23–35)] were included. Patients received lumacaftor/ivacaftor and/or elexacaftor/tezacaftor/ivacaftor for a median of 43 months (range, 15–123) until last assessment (T2). The prevalence of CFLD decreased from 15 (26.3%) at T0 to 8 (14.0%) at T2 (*p* = 0.016), and no new cases of CFLD were observed. Median LSM decreased from 6.2 (IQR 4.9–8.0) to 5.0 kPa (IQR 4.1–6.2) in the overall cohort (*p* < 0.001) and from 10.2 (IQR 6.8–13) to 6.2 kPa (IQR 5.0–12.4) in the CFLD subgroup (*p* = 0.025). Mild, transient fluctuations in liver enzymes occurred in 26.3% of patients. In conclusion, adults with CF receiving long-term CFTR modulators, showed improvement of liver disease assessed by ultrasonography and transient elastography. At the last assessment, half of the patients no longer met the criteria for CFLD.

## 1. Introduction

Cystic fibrosis (CF) is an autosomal recessive genetic disorder with high prevalence in Caucasian populations [1]. Mutations in the gene encoding CF transmembrane conductance regulator (CFTR), a chloride-conducting transmembrane channel, result in impaired transport of bicarbonate and chloride anions and diminished mucociliary clearance of the airways leading in mucus plugs, chronic infection, and local airway inflammation [2,3]. CF affects several organ systems, including the liver and bile ducts (Cystic Fibrosis-associated Liver Disease—CFLD) [1]. Hepatobiliary involvement increases with age and accounts for about 32% by age 25 [4].

Liver disease in CF may be related to the underlying CFTR defect itself but also to etiologies that are indirectly related to CF, like drug hepatotoxicity, bacterial infections of the liver and/or biliary tract, liver steatosis associated with diabetes mellitus and liver congestion as a cardio-pulmonary complication [5]. CFTR defect in hepatic endothelium and cholangiocytes induces abnormal flow of anions, high viscosity, and biliary obstruction [6]. CFLD includes a broad pattern of liver and biliary disorders which may overlap, i.e., multinodular cirrhosis, focal biliary fibrosis, liver steatosis, porto-sinusoidal vascular disease, sclerosing cholangitis with focal or multiple biliary stenosis, disorder of the gallbladder, etc. [6]. Usually, it is difficult to discriminate if the liver disorder is related to the underlying CFTR defect or is secondary due to extrahepatic or iatrogenic complications [7].

It has been reported that CFLD is the third leading cause of mortality in CF after respiratory failure and complications of transplantation, but most data are on pediatrics [8,9]. The diagnosis of CFLD is important for outcome prediction but is difficult to be documented in its early stages, as it progresses insidiously until advanced, when life-threatening manifestations appear. A combination of diagnostic modalities including physical examination, liver enzymes, and imaging was utilized in the conventional Debray criteria [10]. Liver biopsy is the gold standard for the diagnosis and staging of liver diseases [11,12] but is not widely used in CF population mainly due to patchy pattern of liver lesions and the invasive nature of the procedure. Thus, non-invasive liver fibrosis tests (NITs), mainly liver stiffness measurement (LSM) evaluated by transient elastography (TE), are valuable tools for the identification of CFLD [6,13,14].

Ursodeoxycholic acid has been proposed to improve bile flow and bicarbonate secretion but no clinical benefit was reported to justify its routine use in CFLD [6,7]. Innovative therapies have been approved starting with lumacaftor in combination with ivacaftor (LI) [15,16] and more recently the triple elexacaftor/tezacaftor/ivacaftor (ETI) for patients harboring at least one copy of the DF508del mutation in the CFTR gene [17,18]. These CFTR modulators were reported to improve lung function, decrease CF pulmonary exacerbations, and have extrapulmonary benefits [19]. Lumacaftor, elexacaftor, and tezacaftor act as correctors, helping the misfolded CFTR protein to be processed correctly and reach the cell surface, while ivacaftor acts as a potentiator, enabling the channels to open and chloride ions to flow through the defective channels [6,7]. However, the drug efficacy and tolerability of adults with CFLD need further investigation. In the current study, we explored the impact of LI and/or ETI on adults with CFLD by evaluating longitudinal changes in liver enzymes, ultrasonography, and LSM.

## 2. Materials and Methods

### 2.1. Design of the Study

This is a single center, non-interventional, mixed retrospective—prospective real-world study. Longitudinal data were collected from July 2017 to April 2025. Only patients who were receiving CFTR modulators, either LI and/or ETI were included in the study. Patients with hepatitis B, C, or alcohol consumption more than 30 gr/day for men or more than 20 gr/day for women were excluded. No patient was obese. No patient with decompensated liver disease received CFTR modulators. Of 66 patients who were first evaluated, 9 were excluded. Seven patients were excluded due to ineligible mutations, one discontinued treatment and another had decompensated liver disease with ascites. Thus, 57 patients with CF diagnosed with sweat chloride concentration exceeding 60 mmol/L and confirmed by a genetic test were included.

The study had a retrospective followed by a sequential prospective component. Existing data from medical records stored in the center or databases were recorded (retrospective component). The same subjects were followed forward, and additional data and outcomes were collected (prospective component). The first recording of the study (baseline) was the initiation of a CFTR-modulator (baseline) (T0) and the last recording, the time of last assessment (T2). The time of prospective study onset is defined as T1. The median duration of the retrospective component was 15 months (IQR 6–44), while that of prospective component was 25 months (24–27). Demographic (age, gender, body mass index [BMI]), clinical examination, laboratory (hemoglobin, white blood cell and platelet counts, AST, ALT, ALP, gammaGT), ultrasound of the upper abdomen, Doppler imaging of splenoportal axis and LSM data were retrospectively (T0–T1) and then prospectively (T1–T2) collected in all patients. The time elapsed between laboratory and LS evaluation was not longer than 3 months. FibroScan^®^ Echosens (Paris, France) was used for LSM. During the prospective part of the study, liver enzymes were tested every 6 months. Nineteen (33.3%) patients started with LI and then changed without interruption to ETI (LI + ETI group), 37 (65%) started with ETI alone (ETI group), and 1 patient continued to LI alone (1.7%) until the end of follow-up. A total of 48 (84.2%) patients who had at least one F508del mutation were first started CFTR modulators while the rest (15.8%) received ETI in the beginning of the treatment as off label use after legal approval by the Greek Pharmaceutical Regulatory Agency. The study protocol was approved by the Hospital Ethical Committee. All patients signed a written informed consent before their inclusion in the study.

### 2.2. CFLD Patients

All patients (N = 57) were evaluated for CFLD by liver enzymes, clinical examination, ultrasound of the upper abdomen, LSM, and other NIT, like FIB-4 and APRI at least three times; at T0, T1, and T2. The CFLD diagnosis, according to the literature [9], was based on Debray criteria [10] at first and then on NITs. Liver ultrasound was considered a key examination for the diagnosis of CFLD in present study, according to Dana et al. [7]. However, abnormal liver enzymes and NIT, especially liver elastography, was also used according to cutoffs recommended in adults by Koh et al. [13]. More specifically, it was designed so that those who had liver stiffness >6.8 kPa and/or APRI > 0.5 and/or FIB-4 > 1.5 could be included in CFLD group [13]. Our diagnostic approach for liver involvement is defined by the algorithm described in the review by Dana et al. [7]. All patients with CFLD were treated with ursodeoxycholic acid until T0. Longitudinal changes in liver enzymes, ultrasonography abnormalities, and LSM are exhibited.

### 2.3. Statistical Analysis

Categorical variables were expressed as count (percentage) and differences over time were compared using the McNemar test. Quantitative variables were expressed as median and interquartile ranges (IQR).

The Wilcoxon signed-rank test for two related samples was used to evaluate changes in variables in the same subjects over two time points (T0 and T2 or T1 and T2). All statistical analyses were performed by the statistical package SPSS (version 21; SPSS Inc., Chicago, IL, USA).

## 3. Results

### 3.1. Total Patients

Fifty-seven patents with CF [56.1% male, median age at diagnosis 6 months [Interquartile range (IQR) 1.5–24] months, median age at baseline (T0) 26 (23–35) years] were included. All but two patients (14 and 16 years old) were adults at T0. Forty-eight patients (84.2%) have at least one copy of the F508del mutation. A total of 19 carried two (33.3%) and 29 (50.9%) one copy (Table 1).

The median (IQR) period of drug administration was 43 (35–67) months in total, 44 (29.5–60) for LI, 39 (31–43.5) for ETI and 80 (63–102) for LI+ETI (Table 1). All patients had received LI and/or ETI for at least 15 months until the last assessment (T2). Forty-eight patients had received treatment before T1 and the remaining nine around that time point. Body mass index and hemoglobin values showed a statistically significant increase in T2 compared to T0 (Table 2). Platelet count showed a statistically significant decrease and total bilirubin an increase in T2 compared to T0 but the values were within normal limits. No statistically significant differences were observed in aminotransferases, ALP or gammaGT values between T0 and T2.

**Figure 1:**
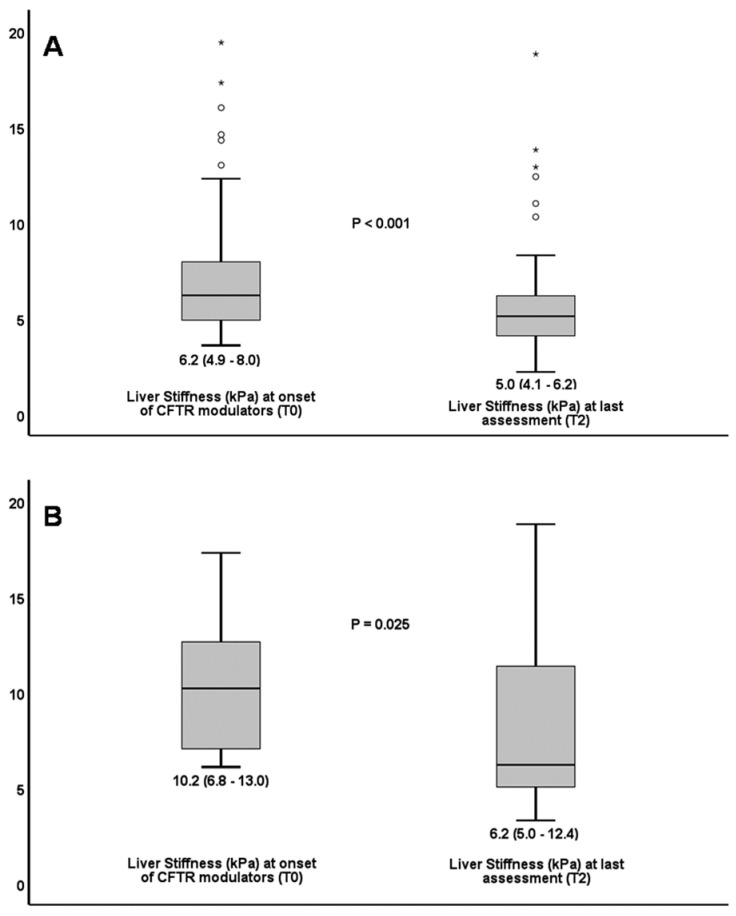
Decline in liver stiffness from treatment onset to last assessment in all patients (**A**). Decline in liver stiffness from treatment onset to last assessment in patients with cystic fibrosis liver disease (**B**). Circles (o) denote mild outliers i.e., values between 1.5 and 3 times above the upper quartile (Q3). Asterisks (*) denote far outliers i.e., values more than 3 times above Q3.

Median LSM decreased from 6.2 kPa (IQR 4.9–8.0) at T0 to 5.0 kPa (IQR 4.1–6.2) at T2 (*p* < 0.001) (Table 2, Figure 1A). It is important that Liver Stiffness (LS) continued to decline during the prospective period of the study, long after initiation of treatment [from 5.4 (4.6–6.6) at T1 to 5.0 (4.1–6.2) at T2 (*p* = 0.009)]. AST to Platelet Ratio Index (APRI) values were more than 0.5 in 2 (both included in CFLD group) at T0 and in nobody at T2. Fibrosis-4 Index (FIB-4) did not help to diagnose CFLD, as it never reached a value of more than 1.5 at any time of the study.

Fifteen (26.3%) patients at T0, and 8 (14.0%) at T2 were characterized as CFLD (*p* = 0.016) (Table 3). Seven out of fifteen demonstrated mild or normal ultrasonography findings (Table 4) and low LSM < 6.85 kPa (Table 3) at the last assessment (T2) and were declassified from CFLD group. No new patient developed CFLD and no patient had liver-related complications during the total study period.

Mild fluctuations in liver function tests (2–3 times the upper limit of normal) occurred in 15 patients (26.3%) in the beginning of ETI treatment but did not require discontinuation of therapy. Only one patient with CFLD received the drug initially in a reduced dose and then returned to the recommended dose.

### 3.2. CFLD Patients

A total of 11 out of 15 (73.3%) who were classified as CFLD at baseline were male. From those with CFLD, 14 had at least one copy of the F508del mutation (Table 3). Liver, biliary, and spleen imaging modalities at T0 and T2 are shown in Table 4. One patient (#9) classified as CFLD from baseline to the end of the follow-up despite normal liver ultrasonography, LSM, or APRI at T2. However, she had persistently elevated liver enzymes × 3–5 times the upper normal limit.

Based on findings in ultrasonography and LS values, eight patients (#1, #4, #10, #11, #12, #13, #14, #15) had advanced chronic liver disease and/or portal hypertension at T0 and seven of them remained CFLD at T2 (#1, #10, #11, #12, #13, #14, #15) with LSM >6.85 and/or ultrasonography findings consistent with liver disease. The remaining (#4) showed a decrease in LSM less than 6.85 and normalization of ultrasonography findings (Table 3 and Table 4). Two of patients with advanced chronic liver disease (#1 and #12) both at T0 and T2 had clinically significant portal hypertension with oesophageal varices diagnosed at the upper GI endoscopy.

LSM in CFLD patients decreased from 10.2 kPa (6.8–13) to 6.2 (5.0–12.4) kPa at T0 and T2, respectively (*p* = 0.025) (Figure 1B). No patient experienced ascites, variceal hemorrhage or died due to liver complications during the total study period.

## 4. Discussion

In this study, we found that adult patients with CF under treatment with CTFR modulators for 15 to 123 months showed a significant regression in liver fibrosis throughout the total study period. Moreover, 26.3% of patients were diagnosed with CFLD in the initiation of treatment but only 14.0% in the last assessment while no patient developed de novo CFLD during treatment.

Liver involvement in CF is challenging to diagnose, as most patients are asymptomatic and may present with normal liver enzymes until advanced disease develops (Σφάλμα! Δεν έχει οριστεί σελιδοδείκτης, [7,19]). Hence, liver disease may be underdiagnosed and underestimated. The use of liver biopsy is considered essential to differentiate cirrhotic from non-cirrhotic portal hypertension and to diagnose the liver disease pattern, stage, and progression. However, it did not reach sufficient consensus due to its invasive nature and to sampling errors in the setting of focal lesions of CFLD [6]. Therefore, some investigators recommended dual-pass percutaneous liver biopsy to avoid sampling errors [12,20]. Non-invasive biomarkers were added to the conventional assessment to identify all patients with liver disease [12,21]. Transient or ShearWave elastography (SWE) are considered the most valuable tools for estimating liver fibrosis and have the advantage of repeated measurements during liver disease course to assess fibrosis progression or regression [22]. However, LSM may misjudge liver disorders like steatosis, cholangiopathy and porto-sinusoidal vascular disease common in CFLD. Regarding liver steatosis, controlled attenuation parameters or magnetic resonance proton density fat fraction can be used to identify it. Injury of intra-hepatic ducts manifesting as single stenosis or multiple stenoses are difficult to diagnose, and magnetic resonance cholangiography is needed. Finally, porto-sinusoidal vascular disease is usually underestimated by all the above methods and liver histology is required to provide the evaluation of that entity [6,7].

Other biomarkers such as APRI or FIB-4 have been widely studied in various forms of liver disease but their usefulness in CFLD is less established [6,7].

In the current study, LSM confirmed CFLD at baseline and demonstrated significant regression of fibrosis at the end of follow-up after long-term CFTR modulator therapy. FIB-4 did not help to diagnose CFLD, as it never reached a value more than 1.5. On the other hand, the conventional criteria should not be overlooked as four (26.6%) patients with findings consistent with CFLD in ultrasonography had LSM less than 6.85 kPa at baseline. It seems, therefore, that diagnostic methods should be used in parallel and in a complementary way and it is wise for the clinician to use a variety of diagnostic methods to accurately identify all patients with CFLD.

CFTR modulators act in extra-pulmonary epithelium by improving its function. Regarding gastrointestinal involvement, an improvement in pancreatic disease by improving glycaemic control [23] and a modest improvement in gastrointestinal symptoms and markers of inflammation after a short period of 6 months of ETI administration was demonstrated [24]. Theoretically, CFTR modulators could improve abnormal CFTR in cholangiocytes and liver endothelium by enhancing bicarbonate secretion and stimulating bile flow [25]. However, in clinical practice, the results are controversial regarding CFLD. In the United States CF Foundation Patient Registry, no difference in aminotransferase values were reported in a mean duration of 20 months of ETI treatment [26]. In another study of 6 month-duration including children and young adults, no difference in liver fibrosis assessed by SWE was observed [27] while in another, in adolescents or young adults, where liver fibrosis was assessed by transient elastography, reduction in liver stiffness was reported [28]. However, most data are on pediatrics and the effect of CFTR modulators on adults with CFLD is scarce. No changes in liver fibrosis outcomes apart from APRI were seen in a study (mean duration of 21 months) of 74 adults [29]. It is reasonable that adults have more frequently advanced liver fibrosis compared to children, resulting in irreversible liver damage. In that case, treatment focuses on stopping or slowing its progression and preventing complications.

In this study, we have evaluated longitudinal liver-related outcomes for a long period of time, i.e., 15–123 months of LI+ETI or ETI alone in adults. We incorporated a variety of methods to capture liver disease progression or regression over time including laboratory, imaging modalities, and surrogate markers of fibrosis. We found both an essential reduction in LSM in total cohort as well as in those with CFLD. Ultrasound abnormalities and liver fibrosis markers consistent with CFLD, when present at treatment initiation, normalized or significantly improved at the last evaluation in almost half CFLD patients (7 out of 15) in a way that they could not be classified as CFLD. The most important finding was that no new patient developed CFLD during the treatment period. In addition, no CFLD patient developed any liver-related complications during the long study period. However, seven out of eight patients who remained CFLD at last assessment (despite the improvement in LSM) may have long-term chronic liver disease and/or portal hypertension at baseline and two of them had documented clinically significant portal hypertension with oesophageal varices diagnosed by upper GI endoscopy. Our findings support the view that individuals with chronically established liver disease may not completely respond to CFTR modulators even after long-term treatment. Consequently, treatment may be most beneficial if it is administered at an early liver disease stage. Liver enzymes did not show a reduction longitudinally. It is evident from the literature that liver biochemistry tests fail to accurately evaluate severity of liver disease or predict liver-related outcomes [6].

Importantly, in our study, no patient discontinued treatment due to liver enzyme abnormalities. Mild elevations of liver enzymes were frequent but returned to normal with ongoing treatment and no patient developed severe hepatic complications. This is explained by the fact that our study population were adults at ETI onset and liver enzyme abnormalities caused by CFTR modulators are more frequent in young children [30].

Our study acknowledges strengths. It is a real-word study on adults where the data in the literature are rare. It is a long-lasting study with treatment administration from one to ten years. Moreover, many diagnostic modalities for the evaluation of liver disease have been used longitudinally and no patient was lost from follow-up.

The limitations of the study are related to its retrospective nature as the retrospective period of the study was long without frequent evaluations. For example, during this period, confounding factors (like nutrition, BMI, and inflammation) may have improved overall clinical status. Furthermore, there is a lack of a control group not receiving CFTR modulators. Finally, the study has limited generalizability due to single-center and small CFLD subset.

## 5. Conclusions

In conclusion, this long-term, real-world study of adults receiving CFTR modulators showed improvement of liver disease assessed by ultrasonography and transient elastography. At the last assessment, about half of the patients no longer met the criteria for CFLD. No liver-related complications were observed even in those who remained with liver involvement until the end of follow-up. No new cases of CFLD emerged during the period of the study. Well-designed, long-term, prospective studies are needed to reach safe and accurate conclusions about the impact of CFTR modulators on the natural history of CFLD.

## Figures and Tables

**Table 1:** Demographics, genotypic, and treatment characteristics at baseline in all patients (N = 57).

**Characteristic**	
Age at initiation of treatment (years)	26 (23–35)
Gender (Male%)	32 (56.1)
Median Age of CF diagnosis (months)	6 (1.5–24)
Genotype (N%)	
DF508/DF508	19 (33.3)
DF508/0ther	29 (50.9)
Other	9 (15.8)
Chloride sweat test ≥60 mmol/L(N%)	57 (100)
**Type of treatment (N of patients)**	**Treatment duration in months [median (IQR) (range)]**
LI+ETI (N = 19) *	80 (IQR 63–102) (range 37–123)
LI only (N = 1)	29
ETI only (N = 37)	39 (IQR 31–43.5) (range15–69)
Total duration of CFTR modulators treatment	43 (IQR 35–67) (range 15–123)

LI, lumacaftor/ivacaftor; ETI, elexacaftor/tezacaftor/ivacaftor; * the 19 patients who received LI before ETI had a median LI administration of 44 months (IQR 29.5–60) (range 10–83).

**Table 2:** Comparison of Body Mass Index, laboratory values, and characteristics of liver disease at initiation of treatment and last assessment in all patients (N = 57).

	Onset of CFT Modulators (T0)	Last Assessment (T2)	*p* Value
Body mass index (BMI) (kg/m^2^)	22.4 (19.6–24.1)	23.3 (20.9–26.4)	<0.001
Laboratory values			
Hemoglobin (gr/dL)	14.0 (12.8–15.1)	14.4 (12.95–15.1)	0.033
White blood cell count(×10^9^/L)	8.12 (6.71–9.26)	6.95 (5.88–8.51)	0.065
Platelet count (×10^9^/L)	292 (244–345)	273 (223–311)	0.002
AST (IU/L)	20 (17–26)	21 (18–26.5)	0.301
ALT (IU/L)	23 (16–35)	23 (17–29)	0.786
ALP (Value/ULN)	0.82 (0.64–1.02)	0.82 (0.63–1.02)	0.245
gammaGT (IU/L)	14 (10–24.5)	14 (10–19.5)	0.42
Total Bilirubin (mg/dL)	0.48 (0.31–0.67)	0.63 (0.43–0.95)	<0.001
Non-invasive Fibrosis markers			
Liver Stiffness (kPa)	6.2 (4.9–8.0)	5.0 (4.1–6.2)	<0.001
APRI > 0.5 (N%)	2 (3.5%)	0 (0%)	NS
CFLD rate over time			
CFLD (N%)	15 (26.3%)	8 (14.0%)	0.016

CFLD, cystic fibrosis liver disease; the BMI, laboratory and liver stiffness values are expressed in median (IQR).

**Table 3:** Demographic, genotypic, and treatment characteristics of patients with CFLD. Liver stiffness evaluation in different time points of the study and classification according to CFLD at the onset of treatment (T0) and last assessment (T1).

Pt #	Genotype	Age at Treatment Onset (years)	Gender	Total Treatment Duration (months)	ETI Duration (months)	LSM (kPa) at Treatment Onset (T0)	LSM (kPa) at Initiation of Prospective Period (T1)	LSM (kPa) at Last Assessment (T2)	CFLD at Treatment Onset	CFLD at Last Assessment
1	DF508/DF508	14	M	116	40	10.4	19.7	18.8	YES	YES
2	DF508/DF508	16	M	72	36	7.3	6.1	4.8	YES	NO
3	DF508/G542X	19	F	53	53	7.9	7.5	5.1	YES	NO
4	DF508/DF508	23	M	93	40	9.0	7.6	6.1	YES	NO
5	DF508/DF508	24	M	80	40	6.2	6.6	6.8	YES	NO
6	DF508/621+1G>T	25	M	39	39	6.8	5.0	5.2	YES	NO
7	DF508/C524x	26	M	39	39	6.1	6.1	4.9	YES	NO
8	DF508/W496X	26	M	61	61	6.8	6.8	3.3	YES	NO
9	DF508/1677delTA	27	F	34	34	10.2	5.4	5.0	YES	YES
10	DF508/DF508	29	M	72	42	17.3	6.6	6.2	YES	YES
11	DF508/DF508	30	F	101	44	11.4	10.5	10.3	YES	YES
12	G542x/621+1G>T	31	M	15	15	13.0	13.0	12.4	YES	YES
13	DF508del/621+1G>T	37	M	34	34	16.0	16.0	12.9	YES	YES
14	DF508/G542X	38	M	29	29	12.3	12.3	13.8	YES	YES
15	DF508/N1303K	33	Μ	41	41	14.6	7.9	7.9	YES	ΥΕS

CFLD, cystic fibrosis liver disease; ETI, elexacaftor/tezacaftor/ivacaftor; Two of the patients (#1 and #12) both at T0 and T2 had clinically significant portal hypertension with oesophageal varices diagnosed at the upper GI endoscopy; Patient #9 had persistently elevated liver enzymes × 3–5 times the upper normal limit from baseline to last assessment and was classified as CFLD.

**Table 4:** Findings of ultrasound of the upper abdomen at treatment onset and at the end of follow-up in 15 patients with cystic fibrosis liver disease.

Patient #	(T0) Treatment Onset	(T2) Last Assessment
1	Macronodular margin, coarse liver echotexture. Reduced size of right lobe. Enlargement of caudate lobe. Portal vein diameter 1 cm. Portosystemic collaterals: recanalization of umbilical vein, gastroesophageal varices, left gastric vein varices along lesser omentum, splenorenal shunts. Splenomegaly, spleen diameter 17.2 cm.	Liver enlarged, caudate lobe hypertrophy. Diffuse heterogeneous echotexture. Lobulated parenchymal margin. Splenomegaly, spleen diameter 17.4 cm.
2	Hepatomegaly, heterogeneous echotexture with features of fibrosis, increased echogenicity. Lobulated margin. Small collateral varices at splenic hilum. Splenomegaly, spleen size 15 cm.	Liver homogeneous. Increased echogenicity, consistent with mild fatty infiltration. Dimensions within normal limits.
3	Liver mildly enlarged. Diffuse increased parenchymal echogenicity with heterogeneous echotexture. Gallbladder with anechoic content.	Liver normal size. Mildly increased echogenicity.
4	Hepatic heterogeneous echotexture. Portal vein diameter 1.2–1.4 cm, flattened flow waveform. Gallbladder with cholelithiasis. Borderline splenomegaly, spleen 13 cm. Splenic vein diameter 1.2 cm with flattened flow waveform, consistent with portal hypertension.	Liver normal size, 14.2 cm. No focal lesions. Gallbladder with cholelithiasis. Spleen 12.4 cm. Splenic vein 0.8 cm, normal Doppler waveform. Portal vein diameter 1.1 cm, normal phasic Doppler waveform.
5	Diffuse increased liver echogenicity with heterogeneous echotexture. Mild splenomegaly, spleen 13.1 cm. Portal vein maximum diameter 1.0 cm, normal lumen and normal waveforms. Splenic vein diameter 0.7 cm with normal velocities.	Liver with mild heterogeneous echotexture. Spleen normal size, 11.7 cm. Splenoportal axis normal on triplex.
6	Hepatomegaly, liver size 17 cm. Diffuse increased parenchymal echogenicity with heterogeneous echotexture. Splenomegaly, spleen size 15.8 cm.	Liver normal size, no focal lesions. Spleen of normal size.
7	Liver enlarged, 16 cm. Heterogeneous echotexture due to multiple scattered nodular lesions, largest 2.2 cm. Findings may represent imaging features of focal biliary fibrosis. Multiple microlithiasis in gallbladder.	Liver normal size and echogenicity. Multiple microlithiasis and sludge in gallbladder.
8	Diffuse increased parenchymal echogenicity, fatty infiltration. No focal lesions. Borderline splenomegaly, spleen 13 cm. Splenic vein dilated, maximum lumen diameter 1.7 cm at splenic hilum, normal Doppler waveform.	Liver normal size (11 cm) and echogenicity, no focal lesion.
9	Normal findings	Normal findings
10	Liver enlarged, 15.7 cm. Significant heterogeneity and coarse liver echotexture, granular margin, no focal lesions. Borderline splenomegaly, 14.2 cm.	Liver enlarged, 17.1 cm, mild heterogeneous echotexture. Gallbladder sludge with wall aggregates. Focal anterior wall thickening, 1.32 cm, suggestive of chronic cholecystitis. Spleen enlarged, 15.4 cm.
11	Liver 15 cm, heterogeneous echotexture, mild fatty liver. Splenomegaly, spleen 14.8 cm.	Normal findings, spleen 11 cm.
12	Slightly increased liver echogenicity and coarse granular heterogeneous echotexture with mild focal lobulation of margin, findings suggestive of early cirrhotic changes. Contracted gallbladder. Splenomegaly, 15.6 cm.	Significant hepatic heterogeneous echotexture. Splenomegaly, 14.2 cm. Spectral Doppler: flattened waveform of portal vein, absence of respiratory alterations, findings consistent with portal hypertension.
13	Coarse hepatic echotexture, macrolobulated margin. Portal vein diameter 1.5 cm, periportal echogenic thickening. Gallbladder filled with echogenic material, Spleen 13.1 cm.	Cirrhotic liver with lobulated margin, mild hypertrophy of left and caudate lobes. Mild portal vein dilatation. Borderline splenomegaly, 13.5 cm.
14	Liver heterogeneous echotexture with few small hypoechoic elements in right lobe. Fatty liver infiltration. Hepatomegaly with inferior margin extended by 3 cm. Spleen, 13 cm.	Increased hepatic echogenicity consistent with fatty infiltration. Liver size within normal limits. Gallbladder with sludge. Mild splenomegaly, spleen 13.6 cm.
15	Focal increased liver echogenicity, consistent with fatty infiltration. Contracted gallbladder.	Heterogeneous hepatic echotexture.

Two of the patients (#1 and #12) both at T0 and T2 had clinically significant portal hypertension with oesophageal varices diagnosed at the upper GI endoscopy; Patient #9 had persistently elevated liver enzymes × 3–5 times the upper normal limit from baseline to last assessment and was classified as CFLD.

## Data Availability

The data that support the findings of this study are available from the corresponding author upon request.

## Abbreviations

The following abbreviations are used in this manuscript:

ALP	Alkaline Phosphatase
ALT	Alanine Aminotransferase
APRI	AST to Platelet Ratio Index
AST	Aspartate Aminotransferase
BMI	Body Mass Index
CF	Cystic Fibrosis
CFLD	Cystic Fibrosis-associated Liver Disease
CFTR	Cystic Fibrosis Transmembrane Conductance Regulator modulators
ETI	Elexacaftor/Tezacaftor/Ivacaftor
FIB-4	Fibrosis-4 Index
gammaGT	Gamma-Glutamyl Transferase
GI	Gastrointestinal
Hb	Hemoglobin
INR	International Normalized Ratio
IQR	Interquartile Range
LI	Lumacaftor/Ivacaftor
LS	Liver Stiffness
LSM	Liver Stiffness Measurement
NITs	Non-Invasive Liver Fibrosis Tests
SWE	ShearWave Elastography
TE	Transient Elastography
